# Assessment of selected parameters of nutritional status in older people with frailty syndrome — a cross-sectional study

**DOI:** 10.1017/jns.2025.10029

**Published:** 2025-08-29

**Authors:** Marta Muszalik, Agnieszka Kudanowska, Grażyna Puto, Kornelia Kędziora-Kornatowska

**Affiliations:** 1 Department of Geriatrics, Faculty of Health Sciences, Nicolaus Copernicus University in Torun, Collegium Medicum in Bydgoszcz, Bydgoszcz, Poland; 2 Uniwersytet WSB Merito, Gdańsk, Poland; 3 Institute of Nursing and Midwifery, Faculty of Health Sciences, Jagiellonian University Medical College, Krakow, Poland

**Keywords:** ADL, BMI, Frailty syndrome, GDS, IADL, MNA, MMSE, Nutritional status, Older adults, SHARE-FI, WHR, ADL, Activity of Daily Living, IADL, Instrumental Activities of Daily Living, SHARE-FI, Survey of Health, Aging, and Retirement in Europe, GDS, Geriatric Depression Scale, MMSE, Mini Mental State Examination, MNA, Mini Nutritional Assessment, CGA, Comprehensive Geriatric Assessment, BMI, Body Mass Index, WHR, Waist-to-Hip Ratio

## Abstract

This study aimed to assess the relationship between selected parameters of nutritional status and the occurrence of frailty syndrome in older adults by analysing clinical and socio-demographic factors. Methods: The study included 150 community-dwelling participants aged > 60 years who were qualified in the medical centre. The following research tools were used: activity of daily living, instrumental activities of daily living, survey of health, aging, and retirement in Europe, geriatric depression scale, mini mental state examination (MMSE), anthropometric measurements, mini nutritional assessment (MNA), body composition measurements, and biochemical blood tests. Results: The study included 150 people over 60 years of age (mean age 76.2/SD 4.9), including 104 women and 46 men. Participants in the frail group were significantly older (KW-H: P < 0.001) and had a higher level of depression (P = 0.008), whereas on the MMSE scale, they achieved a lower result (P < 0.001) than those in the non-frail and pre-frail groups. People in the frail group had significantly lower levels of nutritional status (P < 0.001) according to the MNA scale, assessment of basic activities in everyday life (P = 0.005), complex activities of everyday life (P < 0.001), hand grip strength of the right hand (P = 0.038) and left hand (P = 0.028), and energy drop (P < 0.001). They were also characterised by difficulties walking (P < 0.001), less frequent physical activity (P < 0.001), loss of appetite (P < 0.001), and weight loss more often (P < 0.05). Conclusion: Advanced age, a greater number of diseases, worse functional and mental performance, and differences in nutritional status and body composition were observed in people with frailty syndrome.

## Introduction

Frailty is characterised by a decrease in the functional reserve of the body and an increased sensitivity to stress. Consequently, it causes unfavourable consequences, such as physical disability, exhaustion and weakness, feelings of fatigue, slower gait, reduced physical activity, weight loss, and increased risk of falls, hospitalisation, and mortality. Progressive failure of physiological systems and a participant’s nutritional status seem to play key roles in the pathogenesis of frailty syndrome.^(1)^ Owing to the multifactorial aetiology, several definitions and assessment methods have been developed, the most frequently used of which are the Fried Frailty Score (Phenotype Score) and the broader Frailty Index (Deficit Accumulation Index).^(2)^

According to the phenotypic model of Linda Fried *et al.*, frailty syndrome is a ‘physiological syndrome characterized by reduced reserves and resistance to stressors, resulting from the accumulation of reduced efficiency of various physiological systems, which in turn leads to susceptibility to unfavourable consequences’. Frailty is characterised by the presence of at least three factors: decreased muscle strength, walking speed and physical activity, unintentional weight loss, and exhaustion.^(1)^

Factors contributing to the occurrence of frailty include age, female sex, low physical activity level, and low level of education.^(3)^ Many frail older people are at risk of malnutrition and consequently poor health. Improving the quality of nutrition in this group is one factor in reducing the risk of frailty. Preventing frailty is a global challenge due to the growing elderly population.^(4)^ Malnutrition among older people is common and depends on their place of residence or economic situation.^(5)^ A link between malnutrition and loss of muscle and fat mass, and thus loss of muscle strength, is likely, but it is not yet clear whether malnutrition is a direct causative factor.^(6)^

The nutritional status of the body consists of structural parameters (e.g. body weight, height, amount of fat tissue, muscle tissue, and so on) and biochemical, which depend on the quality of the diet. As there is no single universal way to assess nutritional status, various methods, including nutritional interviews, anthropometric examinations, and clinical and biochemical examinations, are recommended. A comprehensive assessment of nutritional status is important for every individual assessment.^(7)^

Older people are susceptible to developing eating disorders and, consequently, malnutrition. Malnutrition is the unconscious loss of weight, and consequently, a gradual decline in health, increased use of health services, and ultimately, increased mortality. Older individuals are predisposed to malnutrition, especially if they have chronic mental or physical diseases.^(8)^

This study aimed to explore the relationships between selected nutritional status parameters and the occurrence of frailty in older adults by analysing clinical and socio-demographic factors.

## Methodology

### Study design and participants

The study was conducted from October 2017 to March 2020 in a group of 150 community-dwelling participants 60 years and over of age, whose was invited to participate in the research in the medical centre. This study was conducted in accordance with the guidelines of the Declaration of Helsinki, and all procedures involving human subjects/patients were approved by the Bioethics Committee of Nicolaus Copernicus University in Toruń at Collegium Medicum. Ludwik Rydygier Bydgoszcz KB 336/2017. Written informed consent was obtained from all the participants.

Each participant was informed about the purpose and course of the study, about the possibility of resigning at any stage, and gave written informed consent to participate in the study. The inclusion criteria for the study were age ≥ 60 years and health conditions, which allowed informed consent to participate. The exclusion criteria were as follows: neurological diseases, Parkinson’s disease, a history of stroke within the previous year, conditions after surgical and orthopaedic surgeries for less than 6 months, exacerbations of chronic diseases, conditions that make it impossible to analyse body composition (pacemaker, endoprostheses), serious cognitive disorders, mental illness, and severe depression.

The average age was 76.2 (SD 4.9) years, and 104 women (69.3%) and 46 men (30.7%) were included in the study. Most of the respondents were married and lived in cities with their families. Most had secondary education. Almost half of the respondents considered their economic situation satisfactory. Detailed data are presented in Table 1.

**Table 1.** Characteristics of the study group (N = 150)

Age	Mean /SD	76.2/4.9	
		N	%
Sex	Female	104	69.3
	Male	46	30.7
Marital status	Single	23	15.3
	Free	39	26
	Married	88	58.7
Place of residence	Village	29	19.3
	City	121	80.7
Dwelling	Living alone	54	36
	Living with family	96	64
Education	Professional	35	23.3
	Secondary	66	44
	Higher	49	32.7
Financial situation	Bad	26	17.3
	Sufficient	72	48
	Good	48	32
	Very good	4	2.7
Smoking	Yes	12	8
	Don’t smoke at the moment	56	37.3
	Never smoked	82	54.7
Alcohol	More often than once a month	6	4
	Never	36	24
	No more than 1× a month	104	69.3

To assess socio-demographic factors, an original questionnaire containing questions regarding age, sex, place of residence, marital status, economic situation, level of education, stimulants used (tobacco and alcohol), subjective self-assessment of health, number of medications, diseases, duration of illness, and physical activity was created.

The Activities of Daily Living (ADL) scale, according to Katz, was used to assess the individual’s independence in performing six basic daily activities. A maximum of 6 points was obtained by the participant.^(9)^ The Instrumental Activities of Daily Living (IADL) scale, according to Lawton, was used to assess the individuals’ higher activities.^(10)^ A maximum of 27 points was obtained from the participant.

The Polish adaptation of the Survey of Health, Aging, and Retirement in Europe (SHARE-FI) scale was used to assess frailty syndrome. The data were entered into the calculator, taking into account the individual’s sex, and on the basis of the results, the degree of frailty was assessed and classified into the following subgroups: frail, pre-frail, and non-frail.^(11,12)^

SHARE-FI includes the following criteria: exhaustion, loss of appetite, weakness, walking difficulties, low physical activity.^(11)^

The Mini-Mental State Examination (MMSE) scale, consisting of 30 questions, was used to assess cognitive function. A normal result was considered to be in the range of 27–30 points; 24–26, cognitive impairment without dementia; 19–23, mild dementia; 11–18, moderate dementia; and 0–10, profound dementia.^(13)^ To assess mental state, the Geriatric Depression Scale (GDS) was selected, which consists of 15 questions with ‘yes’ or ‘no’ answers; scores range from 0 to 5 points without depression, 6–10 points with moderate depression, and 11–15 points with severe depression.^(14)^

To assess nutritional status, the full version of the Mini Nutritional Assessment (MNA) scale, which is characterised by high specificity and sensitivity,^(15)^ consists of two parts: a screening part of 6 questions and a participants assessment of 12 questions. This scale includes elements of a nutritional interview, subjective assessment of nutritional status, anthropometric measurements, calf and arm circumference, and calculation of body mass index (BMI). Good nutritional status is in the range of 24–30 points, and the risk of malnutrition is 17–23.5 points; a score of less than 17 points indicates that the individual is undernourished.^(16)^

Anthropometric measurements were also carried out (measuring the circumference of the calf, arm, waist, hips, and height), and body composition analysis was performed using a TANITA 418 BC analyzer.

### Statistical methods

IBM SPSS Statistics 28.0.1 and Stat Soft Statistics 13.1 were used for statistical analysis. Frequencies and descriptive statistics were calculated, tests for compliance with a normal distribution were performed, analyses of variance in the between-subjects design and Kruskal–Wallis tests were performed, and a series of chi-square analyses for cross-tabulations were performed. The analysis of basic descriptive statistics was then subjected to the Shapiro–Wilk test, which examines compliance with the normal distribution of the quantitative variables included in the study, along with tests for normality of distribution. Kramer’s V coefficient was used to observe the relationship that determines the level of dependence between two nominal variables: 0.1, small effect; 0.3, average effect; and 0.5, large effect. Corrected residual values were used for post hoc analysis, where the level of statistical significance was >1.96. The Pearson chi-square test was used to compare multiple nominal values, with a statistical significance level of P < 0.05.

## Results

The subjects were divided into three groups according to the SHARE-FI criteria: frail, pre-frail, and non-frail, each, 50 people. However, sex did not differ significantly between the study groups (Chi2: P > 0.05). Participants in the frail group were significantly older than those in the other groups (KW-H, P < 0.001). The mean age of the non-frail, pre-frail, and frail groups was 74 ± 5.03 years; that of the pre-frail group was 75 ± 4.71 years; and 80 ± 2.86 years. The post hoc analysis revealed significant differences (KW-H: P = 0.00) between the frail, non-frail, and pre-frail groups. The groups did not differ significantly in terms of marital status, presence of family, education, or economic status. However, there was a significant difference in the place of residence. Pre-frail participants lived in rural areas (Chi2: P < 0.001; Kramer’s V: 0.344; RS > 1.96).

Detailed results are presented in Table 2.

**Table 2.** Socio-demographic factors in the study groups (N = 150)

		FRAIL		PRE-FRAIL		NON-FRAIL		
		n	%	n	%	n	%	
N = 150		50		50		50		P
Sex	Female	33	66	31	62	40	80	P > 0.05
	Male	17	34	19	38	10	20	
Age	Years/SD	80/2.86		75 /4.71		74/5.03		P < 0.001
Marital status	Single	6	12	10	20	7	14	P > 0.05
	Widow/widower	20	40	10	20	9	18	
	Married	24	48	30	60	34	68%	
Place of residence	Village	3	6	19	38	7	14	P < 0.001
	City	47	94	31	62	43	86	
Dwelling	Alone	23	46	17	34	14	28	P > 0.05
	With family	27	54	33	66	36	72	
Education	Professional	10	20	11	22	14	28	P > 0.05
	Secondary	25	50	16	32	25	50	
	Higher	15	30	23	46	11	22	
Financial situation	Bed	5	10	9	18	12	24	P > 0.05
	Sufficient	24	48	23	46	25	50	
	Good	19	38	17	34	12	24	
	Very good	2	4	1	2	1	2	

Restrictions in food consumption were observed in the frail group (Chi2: P < 0.05; Kramer’s V = 0.29; RS > 1.96). Frail participants more often lost more than 3 kg of body weight in the 3 months preceding the study than the other groups (Chi2: P < 0.001; Kramer’s V = 0.405; RS > 1.96). The pre-frail group took more medications than the other groups. More participants in the frail group than in the non-frail group were taking more than three prescription medications per day (Chi2: P < 0.05; Kramer’s V = 0.246; RS > 1.96). Compared to the other groups, the frail group consumed fewer full meals. The consumption of three meals per day was greater in the non-frail group than in the pre-frail group (Chi2: P < 0.001; Kramer’s V = 0.269; RS > 1.96). Fewer people in the frail group consumed a portion of meat, fish, or poultry daily than those in the pre-frail group (Chi2: P < 0.05; Kramer’s V = 0.202; RS > 1.96). More participants in the frail group reported that they did not consume two or more portions of fruits or vegetables per day (Chi2: P < 0.05; Kramer’s V = 0.271; RS > 1.96). Pre-frail participants had smaller arm circumferences (Chi2: P < 0.001; Kramer’s V = 0.421; RS > 1.96) and frail calf circumferences (Chi2: P < 0.05; Kramer’s V = 0.238; RS > 1.96).

Detailed results are presented in Table 3.

**Table 3.** Differences in parameters of nutritional status of participants divided into groups according to MNA

		FRAIL		PRE-FRAIL		NO-FRAIL		P
		n	%	n	%	n	%	
MNA								
Eating meals	Moderate restriction of food intake	17	34	9	18	3	6	P < 0.05
	No food restrictions	33	66	41	82	47	94	
Weight loss in the last 3 months	Weight loss of more than 3 kg	23	46	11	22	3	6	P < 0.01
	Weight loss between 1 and 3 kg	8	16	9	18	5	10	
	No	8	16	21	42	41	82	
Moving	Can get up from bed or chair	4	8	0	0	2	4	P > 0.05
	Without limits	46	92	50	100	48	96	
Psychological stress or serious illness	Yes	11	22	13	26	10	20	P > 0.05
	No	39	78	37	74	40	80	
Neuropsychological disorders	Severe dementia or depression	0	0	0	0	0	0	P < 0.5
	Mild dementia	13	26	7	14	4	8	
	No psychological disorders	37	74	43	86	46	92	
BMI	BMI < 19	1	2	2	4	0	0	P > 0.05
	19 ≤ BMI < 21	1	2	1	2	0	0	
	21 ≤ BMI < 23	7	14	3	6	0	0	
	BMI ≥ 23	41	82	44	88	50	100	
MNA	Correct nutritional status	15	30	29	58	45	90	P < 0.001
	The threat of malnutrition	32	64	21	42	5	10	
	Malnutrition	3	6	0	0	0	0	
Participants assessment								
Independent living	Yes	49	98	50	100	50	100	P > 0.05
	No	1	2	0	0	0	0	
Takes more than 3 medications a day	Yes	4	8	16	32	13	26	P < 0.05
	No	46	92	34	68	37	74	
Bedsores or skin ulcers	Yes	44	88	44	88	45	90	P > 0.05
	No	6	12	6	12	5	10	
Number of full meals eaten per day	1 meal	0	0	3	6	0	0	P < 0.001
	2 meals	18	3	7	14	3	6	
	3 meals	31	62	39	78	47	94	
At least one serving of products per day	Yes	42	84	42	84	46	92	P > 0.05
	No	8	16	8	16	4	8	
Two or more legumes or eggs per week	Yes	32	64	35	70	40	80	P > 0.05
	No	18	36	15	30	10	20	
A portion of meat, fish or poultry every day	Yes	33	66	42	84	43	86	P < 0.05
	No	17	34	8	16	7	14	
Two or more fruits or vegetables a day	Yes	33	66	45	90	46	92	P < 0.05
	No	17	34	5	10	4	8	
Number of cups/glasses drinks consumed per day	Less than 3	12	24	0	0	1	2	P < 0.001
	3–5	19	38	18	36	10	20	
	over 5	19	38	32	64	39	78	
Nutrition method	Requires assistance while eating	0	0	0	0	0	0	P > 0.05
	He eats meals on his own but with some difficulty	3	6	0	0	1	2	
	He eats meals on his own without any problems	47	94	50	100	49	98	
Self-assessment of nutritional status	He claims to be malnourished	0	0	0	0	0	0	P < 0.001
	Is not sure about his/her own nutritional status	39	78	20	40	8	16	
	He sees no problem with his own nutritional status	11	22	30	60	42	84	
Your own assessment of your health status	Not so good	17	34	7	14	1	2	P < 0.001
	He can’t judge	22	44	14	28	4	8	
	Just as good	10	20	23	46	28	56	
	Better	1	2	6	12	17	34	
Half-length arm circumference (MAC) in cm	MAC < 21	1	2	4	8	0	0	P < 0.001
	21 ≤ MAC ≤ 22	15	30	2	4	3	6	
	BMI > 22	34	68	44	88	47	94	
Calf circumference (CC) in cm	CC < 31	15	30	6	12	5	10	P < 0.05
	CC ≥ 31	35	70	44	88	45	90	
MNA	Correct nutritional status	3	6	26	52	45	90	P < 0.001
	Risk of malnutrition	47	94	24	48	5	10	
Average hand grip strength/SD	Right	24.62/ 10.91		29.59/ 11.74		27.55/8.54		P < 0.05
	Left	23.10/ 11.88		27.61/ 11.35		26.24/8.23		P < 0.05

Participants with frailty syndrome had the highest body weight, while those in the pre-frail group had the lowest (P < 0.05). The highest BMI was found in frail participants, whereas the lowest BMI was found in pre-frail (P < 0.05). The percentage of fat and visceral adipose tissue (TRFATP) was the lowest among the pre-frail subjects at 31% (P > 0.05). Pre-frail participants had the greatest amount of muscle, lean tissue, calf, and upper arm circumferences. The largest waist and hip circumferences were among frail individuals.

The water content did not differ in the studied groups (P > 0.05).

The detailed results are presented in Table 4.

**Table 4.** Body composition analysis according to study groups (N = 150)

	FRAIL		PRE-FRAIL		NON-FRAIL		*P*
	Mean	SD	Mean	SD	Mean	SD	
Height (cm)	163.3	8.8	164.0	8.1	163.8	7.1	*P > 0.05*
Body weight (kg)	75.9	12.4	70.4	11.4	75.8	10.4	*P > 0.05*
BMI (kg/m^2^)	28.5	4.5	26.2	3.9	28.3	4.0	*P > 0.05*
Fat content (%)	35.0	8.6	32.1	8.6	36.5	6.6	*P > 0.05*
Fat mass (kg)	27.1	8.6	23.1	8.0	27.9	7.3	*P > 0.05*
Muscle mass (kg)	46.5	7.9	45.1	7.4	45.5	6.4	*P > 0.05*
Muscle content (%)	61.8	8.3	64.6	8.2	60.4	6.4	*P > 0.05*
FFM (KG)	48.9	8.2	47.4	7.7	47.9	6.6	*P > 0.05*
%FFM	0.6	0.1	0.7	0.1	0.6	0.1	*P > 0.05*
TBW Water mass (KG)	35.7	5.9	34.7	5.6	35.0	4.9	*P > 0.05*
Water content (%)	48	6	50	6	47	5	*P > 0.05*
TRFATP (%)	33.9	10.8	31.0	9.2	34.4	6.8	*P > 0.05*
TRFATM	14.5	4.8	12.5	4.6	14.4	3.9	*P > 0.05*
Circumference arm	24.5	2.9	26.4	3.8	27.0	3.2	*P < 0.001*
Circumference calves	33.2	3.0	34.9	4.1	34.9	4.1	*P < 0.05*
Waist circumference	95.2	11.9	93.1	9.1	92.5	8.2	*P > 0.05*
Hips circumference	100.8	9.0	98.9	8.8	103.3	8.4	*P > 0.05*
WHR	0.9	0.1	0.9	0.1	0.9	0.1	*P < 0.05*

*FFM,* fat-free body weight*; TBW,* water in the body composition*; TRFAT,* visceral fat*; WHR,* hip-to-waist circumference ratio.

The frail group had a significantly greater risk of depression (P = 0.008). The MMSE score was significantly lower (P < 0.001) in the frail group than in the non-frail and pre-frail groups. Additionally, people in the frail group had a significantly lower nutritional status (P < 0.001) than those in the non-frail group.

The results are presented in Table 5.

**Table 5.** Results of variance analyses of GDS, MNA, and MMSE results

	FRAIL (n = 50)		PRE-FRAIL (n = 50)		NON-FRAIL (n = 50)				
	*M*	*SD*	*M*	*SD*	*M*	*SD*	*F*	*P*	η^2^
GDS	4.88	3.11	3.4	2.98	3.3	2.34	489	0.008	0.06
MNA	21.53	2.43	24.31	2.96	27.36	2.22	64.96	<0.001	0.47
MMSE	26.2	2.32	26.58	2.45	28.64	1.88	17.31	<0.001	0.19

M, average; SD, standard deviation; F, ANOVA test result; P, significance of the ANOVA test; η^2^, eta squared (strength of effect).

Significant differences were observed in the assessment of basic ADL (P = 0.005), IADL (P < 0.001), grip strength in the right hand (P = 0.038) and left hand (P = 0.028), and systolic blood pressure among the non-frail, pre-frail, and frail groups. To determine which groups differed from each other, post hoc analysis was performed. The results of this analysis revealed that people in the frail group had a significantly lower assessment of basic activities in everyday life (P = 0.005, effect size ε^2^ = 0.07)) and an average left-hand grip (P = 0.028, ε^2^ = 0.04) in the first and second measurements, respectively, than in the pre-frail and non-frail groups.

The results are presented in Table 6.

**Table 6.** Results of Kruskal–Wallis tests of selected assessment parameters in the groups

	FRAIL (n = 50)		PRE-FRAIL (n = 50)		NON-FRAIL (n = 50)				
	*M*	*SD*	*M*	*SD*	*M*	*SD*	χ^2^	*P*	ε^2^
ADL	5.58	0,61	5.84	0.37	5.88	0.33	10.47	0.005	0.07
IADL	21.80	2.76	22.74	1.72	23.66	1.02	19.07	<0.001	0.13
Hand grip strength: right	24.87	10.93	29.58	11.98	27.71	8.71	6.56	0.038	0.04
Hand grip strength: left	23.18	12.28	27.65	12.20	26.33	8.35	7.17	0.028	0.04
Diastolic blood pressure	81.98	13.11	86.50	15.32	78.90	10.42	6.87	0.032	0.04

M, average; SD, standard deviation; χ^2^, Kruskal–Wallis test result; P, significance of the Kruskal–Wallis test; ε^2^, epsilon-square (strength of effect).

## Discussion

A total of 150 people participated in the study, including 104 women and 46 men, and the average age of the participants in the study group was 76.2 years (SD ± 4.91). Participants in the frail group were significantly older than those in the other groups (P < 0.001).

According to another study, the prevalence of frailty increases with age independently of the assessment instrument, and ranges between 4 and 59% in community-dwelling elderly populations and is higher in women than in men.^(17)^

Analysis of the study group in terms of sex did not reveal any significant differences (P > 0.05). There were 40 women in the non-frail group, 31 in the pre-frail group, and 33 in the frail group.). Of the 104 women, 33 were frail (31.7%), while among 46 men, 17 (36.9%) were frail.

However, gender did not play a role in group assignment probably too small a sample of men (P > 0.05).

In a meta-analysis published in 2021, the authors pointed out differences in assessment depending on the method. The overall prevalence of frailty in studies using physical measures of frailty was 12% (95% CI = 11–13%; n = 178), compared with 24% (95% CI = 22–26%; n = 71) in the deficit accumulation model (using the frailty index, FI). For studies using a FI, the prevalence was also higher in females, 29% (95% CI = 24–35%; n = 34) versus 20% (95% CI = 16–24%; n = 34), for males.^(18)^

In our study, women showed a numerical advantage in each group. An increasing trend towards frailty was observed among widowed people. The majority of individuals with frailty syndrome were single city residents (P < 0.001), whereas rural residents represented the pre-frail group the most. Loneliness was identified as a factor related to frailty, and the study results indicated this relationship.

A 2014 study conducted by Theou and Brothers^(19)^ on a population of 27,527 participants aged 65.5 ± 10.5 years, 55% of which were women in 11 European countries, was based on seven scales: the Edmonton Frail Scale, FRAIL, Groningen Frailty Indicator, frailty phenotype, Tilburg Frailty Indicator, 70-point Frailty Index (FI), and 44-point Frailty Index based on the Comprehensive Geriatric Assessment (CGA). In these studies, the risk of death increased with frailty scores, with women scoring higher than men. A meta-analysis by Gordon *et al.* found that sex differences in frailty and mortality reflect the reported phenomena of women living longer than men despite the addition of health problems. These findings provide a basis for a deeper examination of sex differences in frailty to identify deficits and resources that influence the health and survival of men and women.^(20)^

Many studies have indicated the need to treat frailty as a dynamic, multidimensional condition at three levels, that is, physical, social, and psychological, taking into account many deficits, including diseases, cognitive impairment, psychosocial factors, or abnormalities in biochemical tests.^(21,22)^ Socio-demographic aspects determine the level of frailty at baseline, not the growth rate^(23)^ and in the present study, more pre-frail individuals lived in rural areas than non-frail and frail (P < 0.001). A study by Wu *et al.* ^(24)^ on a group of 2,802 patients revealed a relationship between the place of residence and the occurrence of frailty, indicating a greater percentage of frailty cases in rural areas than in cities.

In our study, the groups did not differ significantly in terms of marital status (P > 0.05). Our study did not reveal any relationship between lifestyle factors, such as smoking, alcohol consumption, or economic situation, as significant factors influencing the occurrence of frailty syndrome, which could have resulted in a narrow group of examined participants not being institutionalised or hospitalised. However, studies conducted by other researchers have indicated a similar lack of a relationship.^(23,25)^

Nutritional status testing is one of the important steps in assessing frailty.^(26)^ Studies have shown a relationship between frailty and nutritional status assessed using the MNA scale.^(27,28)^ In our study, a lower nutritional status score (P < 0.001) was observed in the frail group than in other groups. In our study, the muscle strength of participants in the frail group, as measured with a dynamometer, was significantly lower than that of participants in the other groups, in both the right and left hands (P = 0.038 and P = 0.028, respectively). Reduced muscle strength is associated with an increased risk of falls, loss of mobility and independence, and greater risk of institutionalisation.^(29)^ A significantly lower appetite was observed in the frail group than in the other groups (P < 0.001).

BMI is used in studies to assess nutritional status, and both the phenotypic definitions of frailty and FI show an increased prevalence of frailty among people with low and high BMIs. This was demonstrated by Hubbard *et al.* in a population of 3,055 individuals over 65 years of age.^(30)^ Older obese people are more likely to be frail than those with a normal BMI. In a meta-analysis of eight databases (PubMed/MEDLINE, EMBASE, EBSCO, CINAHL, Scopus, Cochrane Library, and Web of Science), conducted by Yuan *et al.* in 2021, a positive relationship was found between abdominal obesity and frailty to clarify the association between frailty and BMI in older people aged ≥60 years living in the community.^(31)^ The results presented in this study revealed that the frail group had a significantly greater BMI, (28.53 kg/m^2^, than the pre-frail group (26.20 kg/m^2^). Assuming a normal range of 22–27 kg/m^2^, according to Lipschiz,^(32)^ these patients can be classified as overweight at 27.01–31.99 kg/m^2^. Bonnefoy *et al.* confirmed that both too low and too high body weights are associated with frailty among older patients.^(28)^

Research results indicate that frailty affects people with both lower and higher BMI. Although obesity is associated with frailty, the distribution of fat tissue may be a more important determinant of frailty, as demonstrated by McCarthy’s 2019 population-based study of 4,568 people.^(33)^ In this study, the frail group had an average BMI of 29.2 kg/m^2^ and a WHR of 0.912, and the pre-frail group had a BMI of 28.3 kg/m^2^ and an average WHR of 0.899. In our study, the WHR in the frail group was 0.95, that in the pre-frail group was 0.94, and that in the non-frail group was 0.9, indicating analogous relationships. In a study by Juan *et al.*, people with a greater waist circumference had a 57% greater risk of frailty than those with a normal waist circumference.^(31)^

In the research on dynapenic abdominal obesity as a risk factor for Metabolic syndrome (MetS-(hypertriglyceridemia, hyperglycaemia, low HDL (high-density lipoprotein), arterial hypertension or body mass index ≥ 30 kg/m^2^) and its components in individuals 50 years of age or older, the authors in the conclusion of their research found that dynapenic abdominal obesity increases the risk of MetS, with a higher IRR (Incidence Ratio Rate) compared to obesity alone. Dynapenic abdominal obesity was defined based on waist circumference (> 102 cm for men and > 88 cm for women) and grip strength (< 26 kg for men and < 16 kg for women). Following the indicated studies, there is a risk that in addition to frailty, our participants may develop MetS.^(34)^

Notably, the MNA scale assesses items that are useful in the diagnosis of malnutrition in people aged 25 years. Valentini *et al.* reported that nutritional status was closely related to the occurrence of frailty and that the MNA score was a good predictor of pre-frail and frail status.^(35)^ Our study revealed a clear relationship between MNA score and the occurrence of frailty syndrome and may support the correct classification of participants into particular groups.

Our study revealed significant differences (P < 0.05) in anthropometric body measurements between the individual groups regarding the calf circumference and arm circumference. Similar results were obtained in a bioimpedance analysis study involving 656 older patients (275 women and 381 men) aged ≥65 years from the Geriatric Department of Zhejian Hospital to investigate the relationship between body composition and the occurrence of frailty in older patients. The authors reported larger waist circumferences and higher fat mass and body fat percentage in frail patients. Non-frail patients have greater arm and calf circumferences and greater amounts of Fat Free Mass (FFM), skeletal muscle, and water, indicating targets for intervention in patients at risk for frailty.^(36)^

The frail group showed a significant relationship with the occurrence of depressed mood according to the GDS, which confirms the observations of other scientists. Studies in older adults have shown that depression and frailty occur in a significant proportion of older adults with frailty, although it is unclear whether depression promotes frailty, frailty promotes depression, or coexists independently. Other studies have shown that 16–35% of frail individuals also experience depression.^(37)^ The average GDS score obtained in our study for the frail group was 4.88, which was significantly different from that of the non-frail group (mean, 3.30) (P = 0.008).

In our study group, the ADL and IADL results were significantly different between the groups, indicating greater limitations in the domain of ADL in the frail group (P = 0.005) than in the other groups. In the case of complex ADL (IADL), the frail group showed greater limitations than the other groups (P < 0.001). Similar indicators were obtained in another study conducted by Ortuno *et al.*, indicating an increasing daily functional disability and the need to develop a reliable system for assessing and monitoring older patients in terms of frailty, falls, loss of independence, and mobility.^(38)^

Participants had different levels of cognitive functioning according to MMSE, but the results obtained among frail participants were the lowest (P < 0.001). The relationship between frailty and cognitive ability remains in the research phase, and there are few studies on cognitive function and frailty in the MMSE. An analysis of the results of a cross-sectional population-based study (a multicenter, nationwide effort to collect data on frailty and aging in Brazil, known as the FIBRA network) was carried out in Ermelino Matarazzo, a poor subdistrict of the city of São Paulo, Brazil, was performed on 384 people over 65 years of age. In Brazil, an attempt was made to determine the relationship between frailty and cognitive functioning assessed using the MMSE. The results revealed that people with frailty had lower MMSE scores (P < 0.001) than those in other groups. These findings suggest that frailty syndrome is associated with poor cognitive function.^(39)^

The participants included in the study reported a significantly greater number of disease entities and a greater number of medications prescribed by a doctor, which is consistent with several published studies.

People in the frail group were more likely to suffer from hypertension (P < 0.05), diabetes (P = 0.078), and other heart diseases (P < 0.05) than those in the non-frail and pre-frail groups. An analysis of the scientific literature revealed that polytherapy is associated with the development of frailty syndrome and may contribute to the development of unfavourable health effects related to the number of falls or adverse effects of medications. Participants in the frail group had a significantly greater number of treated diseases and a greater number of medications taken, were sick longer (P < 0.05), and had a lower subjective assessment of health (P < 0.001). Hubbard *et al.* reported a significant relationship (P < 0.001) between the number of drugs used and frailty or pre-frailty. The subjective assessment of the health status of patients in the frail group was lower than that of the patients in the other groups (P < 0.001).^(30)^

In managing frailty, many aspects should be taken into account regarding diet planning, appropriately selected physical activity, monitoring body weight, preventing undernutrition or overweight, and supplementation of vitamins and minerals. Conducting systematic and comprehensive examinations, especially by the geriatric team, is important to detect and treat older patients, whose characteristic features are comorbidities besides frailty.

The authors of a review on the identification, management, and treatment of frailty included many significant tips in the Clinical Practice Guidelines for Identification and Management of Frailty.

Older people should be tested for frailty using standardised tools; especially gait speed is a recommended outcome measure for frailty assessment. Elderly subjects should be considered pre-frail and offered screening if present with clinical features.^(40)^

Research on frailty is ongoing; hence many discoveries and achievements have emerged, including new biomarkers and biomarker panels for screening and diagnostics of frailty. Work is ongoing on the use of artificial intelligence to identify frailty and research on the different responses to drugs in older people with frailty. Innovative achievements have been noted in many areas of frailty research, including technology-based exercise training, the use of multidimensional interventions, the implementation of person-centred and integrated care, the use of technology to support diagnosis, risk factor assessment, including analysis of transformations between frailty states, modification of clinical guidelines, and the development of potential future treatments.^(41)^

This study has limitations due to the adopted research methodology and was conducted on a group of participants from the community invited to participate in the study. They were permanently active, aware of risk factors related to diseases and their treatment, and engaged in activities aimed at maintaining health, such as physical, intellectual, and social activity. Hospitalised and institutionalised patients were excluded from this study. Expansion of the study group may have affected the results.

The advantages of this study include that a large group of participants (200 participants were screened) was examined according to a uniform and repeatable model, using standard recommended tools for assessing subjective and objective health status. Each participant in our study received all the necessary advice on maintaining health and resulting from the study results. Participants could also ask questions and receive answers. A study on a healthy, active population of older individuals may be the beginning of further research on other groups of seniors to define the risk factors associated with the occurrence of frailty syndrome.

## Data Availability

The datasets used and/or analysed in this study are available from the corresponding author upon request.
